# The Alpha Crucis Carbonate Ridge (ACCR): Discovery of a giant ring-shaped carbonate complex on the SW Atlantic margin

**DOI:** 10.1038/s41598-019-55226-3

**Published:** 2019-12-10

**Authors:** Mascimiliano Maly, Uri Schattner, Francisco José Lobo, Rodolfo Jasão Soares Dias, Raissa Basti Ramos, Daniel de Matos Couto, Paulo Yukio Gomes Sumida, Michel Michaelovitch de Mahiques

**Affiliations:** 1 Oceanographic Institute of the University of São Paulo, São Paulo, Brazil; 2 Dr. Moses Strauss Department of Marine Geosciences, Leon H. Charney School of Marine Sciences, University of Haifa, Haifa, Israel; 3 Instituto Andaluz de Ciencias de la Tierra (CSIC-Universidad de Granada), Armilla, Spain; 4 Institute of Energy and Environment of the University of São Paulo, São Paulo, Brazil

**Keywords:** Geophysics, Geomorphology, Ecology, Ocean sciences

## Abstract

Recently acquired bathymetric and high-resolution seismic data from the upper slope of Santos Basin, southern Brazilian margin, reveal a major geomorphological feature in the SW Atlantic that is interpreted as a carbonate ridge - the Alpha Crucis Carbonate Ridge (ACCR). The ACCR is the first megastructure of this type described on the SW Atlantic margin. The ~17 × 11-km-wide ring-shaped ACCR features tens of >100-m-high steep-sided carbonate mounds protruding from the surrounding seabed and flanked by elongated depressions. Comet-like marks downstream of the mound structures indicate that the area is presently influenced by the northward flow of the Intermediate Western Boundary Current (IWBC), a branch of the Subtropical Gyre that transports Antarctic Intermediate Water. Abundant carbonate sands and gravels cover the mounds and are overlain by a biologically significant community of living and dead ramified corals and associated invertebrates. The IWBC acts as a hydrodynamic factor that is responsible for both shaping the bottom and transporting coral larvae. We contend that the ACCR was formed by upward fluid flow along active sub-surface faults and fractures that formed by lateral extension generated by the ascending movement of salt diapirs at depth. The ACCR provides an important modern and accessible analogue for a seabed carbonate build-up related to sub-surface hydrocarbon systems.

## Introduction

Carbonate mounds constitute outstanding submarine geomorphological features that exhibit semi-circular to elongated morphologies (i.e., carbonate ridges) extending laterally up to tens of kilometres and varying in height from a few metres to hundreds of metres^1^. These features are regarded as giant mounds when their heights exceed 150 m above the surrounding seafloor^2^. Carbonate mounds have been found at depths of up to 1300 m but are most common in the 500–1300 m water depth interval^2–4^.

The best examples of giant carbonate mounds and elongated ridges have been described on the continental margin off Ireland (e.g.^3,5–8^). Other areas where relatively large carbonate mounds (with maximum heights of less than 100 m) have been reported include the Galicia continental margin^9^, the Gulf of Cadiz^10–12^, the northwestern African margin^13^, and the Mediterranean Sea^14^.

In most cases, these carbonate mounds are interpreted as the result of long-term alternation between the growth and demise of cold-water corals (CWCs)^1,2,6^, which have acted as carbonate factories since the Pliocene^1^. Although some studies indicate that seafloor seepage and fluid flow may be involved in their initial evolutionary stages^3,6^ through the formation of a hard substrate^9^, the prevailing view considers their development to be mainly controlled by physical variables such as currents^3^ and the water mass structure^13^. Indeed, their large-scale distribution is spatially correlated with the boundaries of water masses^1,4^, where different mixing processes (such as internal waves and upwelling) may exert an influence^2,12^. After their initial growth, carbonate mounds can be affected by early diagenetic processes^2,10^ or can modify the velocity and direction of bottom currents^1^.

Methane-derived authigenic carbonates (MDACs) form through the microbially mediated precipitation of carbonate that originally formed in the sub-surface^15^. MDACs include several morphological types, such as crests, chimneys, concretions, and mounds^16^. The Gulf of Cadiz is a key area where numerous manifestations of MDACs have been reported^17,18^. In clear contrast to CWCs, MDACs exhibit smaller dimensions; for example, the highest MDAC ridges are up to 10 m high and several hundreds of metres long^19,20^. The genesis of these MDACs is related to slow flux seepages and gas venting^15,20^.

Understanding the present mechanisms of mound formation on the seafloor can facilitate the interpretation of inactive mounds that today are buried in the sub-surface^21^.

This study reports, for the first time, the occurrence of a giant, ring-shaped, and quasi-continuous carbonate ridge on the SW Atlantic margin. Here, we aim to understand the regional conditions concerning sub-surface fluid flow and the oceanographic regime that enabled the formation of such an impressive feature.

## Study Area

The study area is located in the SW Atlantic margin of the Santos Basin, off southeastern Brazil, between water depths of 450 and 1250 m.

**Figure 1:**
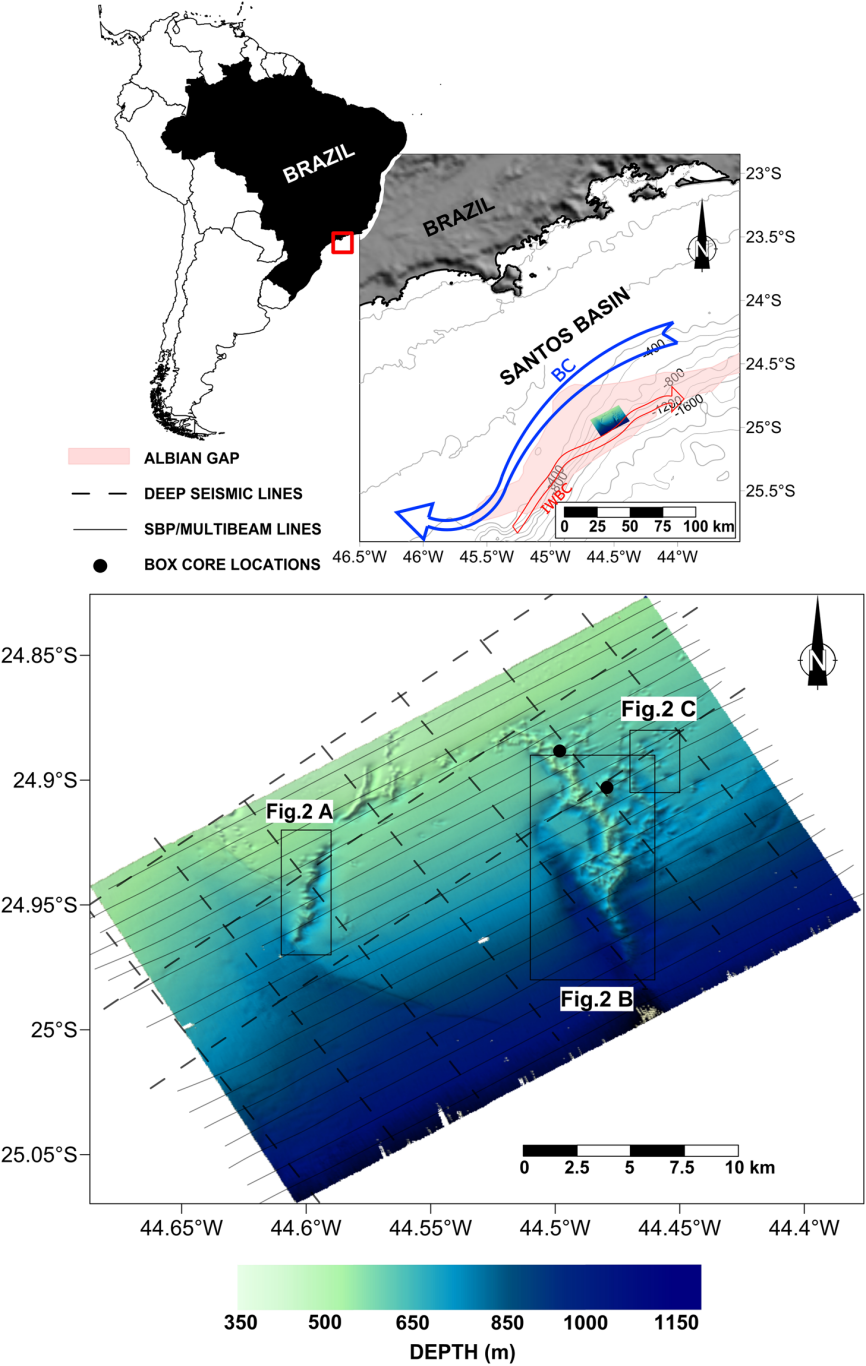
Location map of the Alpha Crucis Carbonate Ridge (ACCR). Thick dashed lines show the location of multichannel seismic lines. Thin continuous lines mark the location of both multibeam and CHIRP acquisition tracks. BC: Brazil Current; IWBC: Intermediate Western Boundary Current. For labels and insets, refer to Fig. 2. The Albian Gap limits are defined as stated by Rodriguez, *et al*.^67^. Map was elaborated with Surfer software, version 13 (GOLDEN SOFTWARE; https://www.goldensoftware.com/products/surfer), and multibeam mosaic was generated using SonarWiz software, version 7.04 (CHESAPEAKE TECHNOLOGIES; https://chesapeaketech.com).

The Santos Basin is a prolific hydrocarbon province, with a nearly 10-km-thick sedimentary column that includes the entire rift, drift, and passive margin evolution from the Barremian to the Neogene^22^. The lower part of the succession includes Aptian clastics that host a rich deep-water hydrocarbon province. The underlying Albian evaporites form sub-surface mounds, domes, walls, diapirs, and more irregular three-dimensional volumes^23^. Most of them are buried tens to hundreds of metres below the present-day ocean floor, while only a few are exposed at the seafloor^22,24–26^. These vertical salt structures underlay most of the Santos Basin and are missing from the 25-km-wide Albian Gap^27–29^ (the proximal portion of the basin, Fig. 1). Lateral salt migration towards the deeper regions of the basin in the east removed most of the salt from the Gap region^29^. This migration induced lateral extensional stresses in the post-Albian succession overlying the Gap, while contractional salt diapirism prevails further eastwards^27,30,31^.

The post-Albian succession contains pelagic and terrigenous sediments that contain additional hydrocarbon sources^22,32,33^. Their accumulation was strongly influenced by two mountain belts^34^. The Andean orogeny uplifted the sediment sources since the Eocene and promoted progradation across the Santos margin^35,36^. The Serra do Mar mountain range uplifted the SE Brazilian coastline in two main events: from 100–70 Ma and later at 15 Ma. The uplift prevented Andean-derived and other terrigenous sediments from being transported directly to the Santos margin. As a result, since the end of the Paleogene, the sediment input to the basin has mainly been sourced from the ocean bottom currents rather than from adjacent rivers^37,38^.

Two dominant seafloor currents of opposing directions flow through the study area^39–41^. The ~0.55 ± 0.23 m.s^−1^ southwestward flow of the Brazil Current (BC in Fig. 1)^42^ polishes the outer shelf and upper slope of Santos Basin down to 500 m water depth^39,43,44^, producing a relict surface^40^. The BC transports Tropical Water (TW) and South Atlantic Central Water (SACW). Below a transition zone between 500 and 600 m water depth, the Intermediate Western Boundary Current (IWBC in Fig. 1) flows northeastwards between 600 and 1200 m water depth^45,46^. The IWBC transports Antarctic Intermediate Water (AAIW) as a part of the Subtropical Gyre^47^ and plays an important role in the distribution of deep-sea corals in the Atlantic^48^. These two currents are the dominant present-day sediment transport mechanism along the ocean floor off SE Brazil; most of the study area lies below the IWBC flow.

The connection between sub-surface hydrocarbon reservoirs, gas migration, and seafloor seepage in the Santos Basin has been established in several recent studies. These studies reveal approximately one thousand pockmarks and other seafloor depressions at water depths of 300–1000 m^33,41,49^. These mass-deficit fluid escape features are associated with the ongoing diapirism above the prolific Santos hydrocarbon province^25,27,50,51^. However, the occurrence of hydrocarbon-associated carbonate giant mounds has not been reported anywhere along the SW Atlantic margin.

## Results

**Figure 2:**
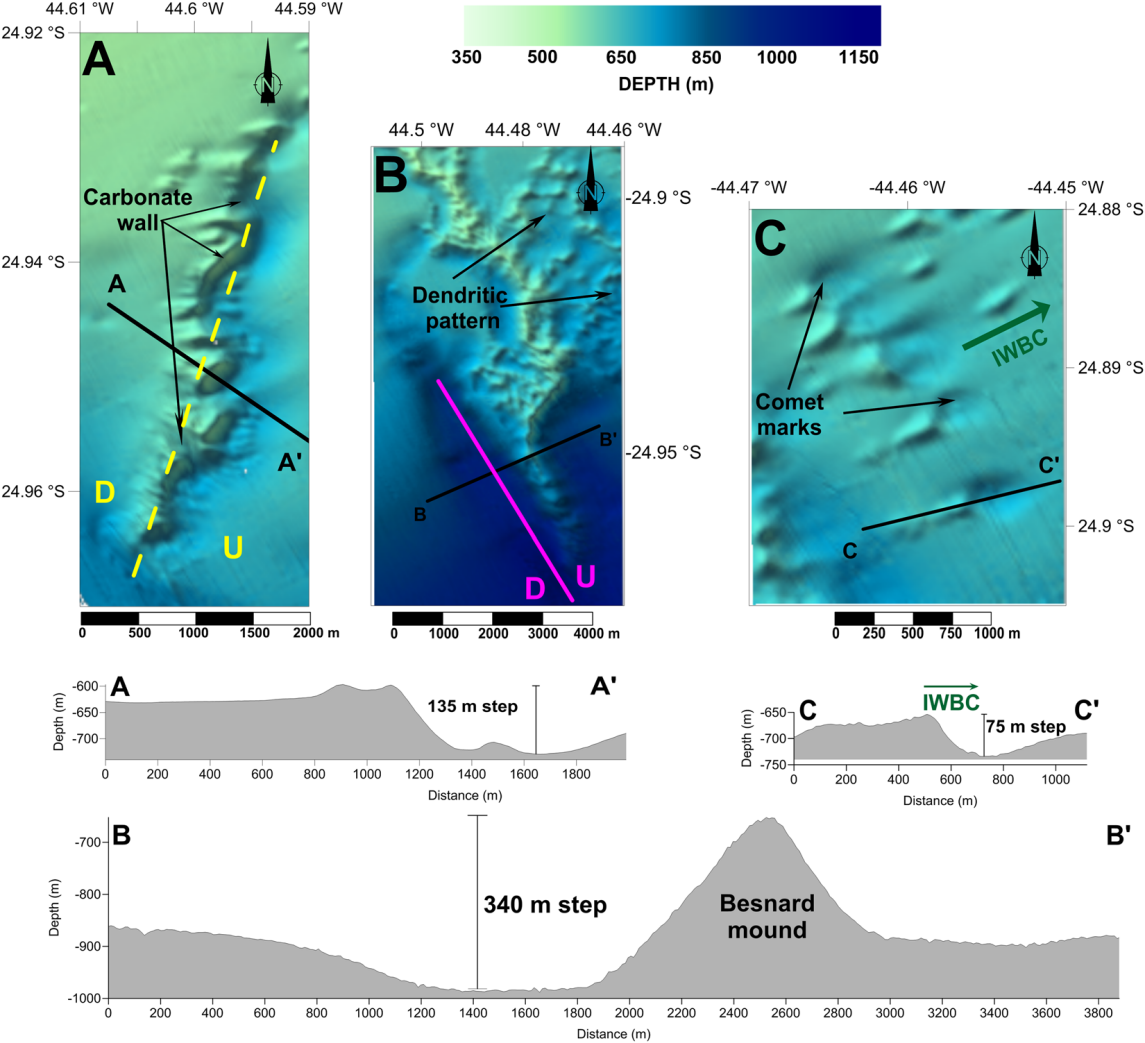
Main morphological features of the ACCR. (**A**) NW narrow ridge sector forming a carbonate wall. Continuous and dashed yellow line shows a normal fault strike. (**B**) ENE wide ridge sector and an indication of the Besnard Mound. The continuous magenta line shows the strike of the west fault (see Fig. 3). (**C**) Elongated depressions (“comet marks”) downstream of the mound structures. The locations of the map insets are indicated in Fig. 1. Interpretation made with SonarWiz software, version 7.04 (CHESAPEAKE TECHNOLOGIES; https://chesapeaketech.com).

Multibeam bathymetric data indicate the presence of a semi-circular, 17.1 by 11.6 km ridge, composed of hundreds of mounds and adjacent depressions on the upper to the middle continental slope of the Santos Basin at water depths between 450 and 1,250 m. The tops of the mounds reach 100 to 290 m above the adjacent seafloor (Fig. 2).

Two main ridge sectors present the most conspicuous mounded features. To the W and NW, the mounds are arranged along narrow, less than 400-m-wide belts (Fig. 2A). The feature is strongly asymmetric and features an ~100-m-deep depression along its eastern flank.

The most impressive mounds are located in the eastern sector; the highest peak of this array is named here the Besnard Mound (Fig. 2B). These mounds are scattered across a wide area and reach heights of ~150 m above the surrounding region. This ridge sector presents eastward-extending dendritic features, which form elongated “comet marks”^52,53^ (Fig. 2C). Two channels flank most of the eastern mounds (Fig. 2B). These channels reach up to 120 m below the adjacent bottom. They become narrower and shallower towards the north.

**Figure 3:**
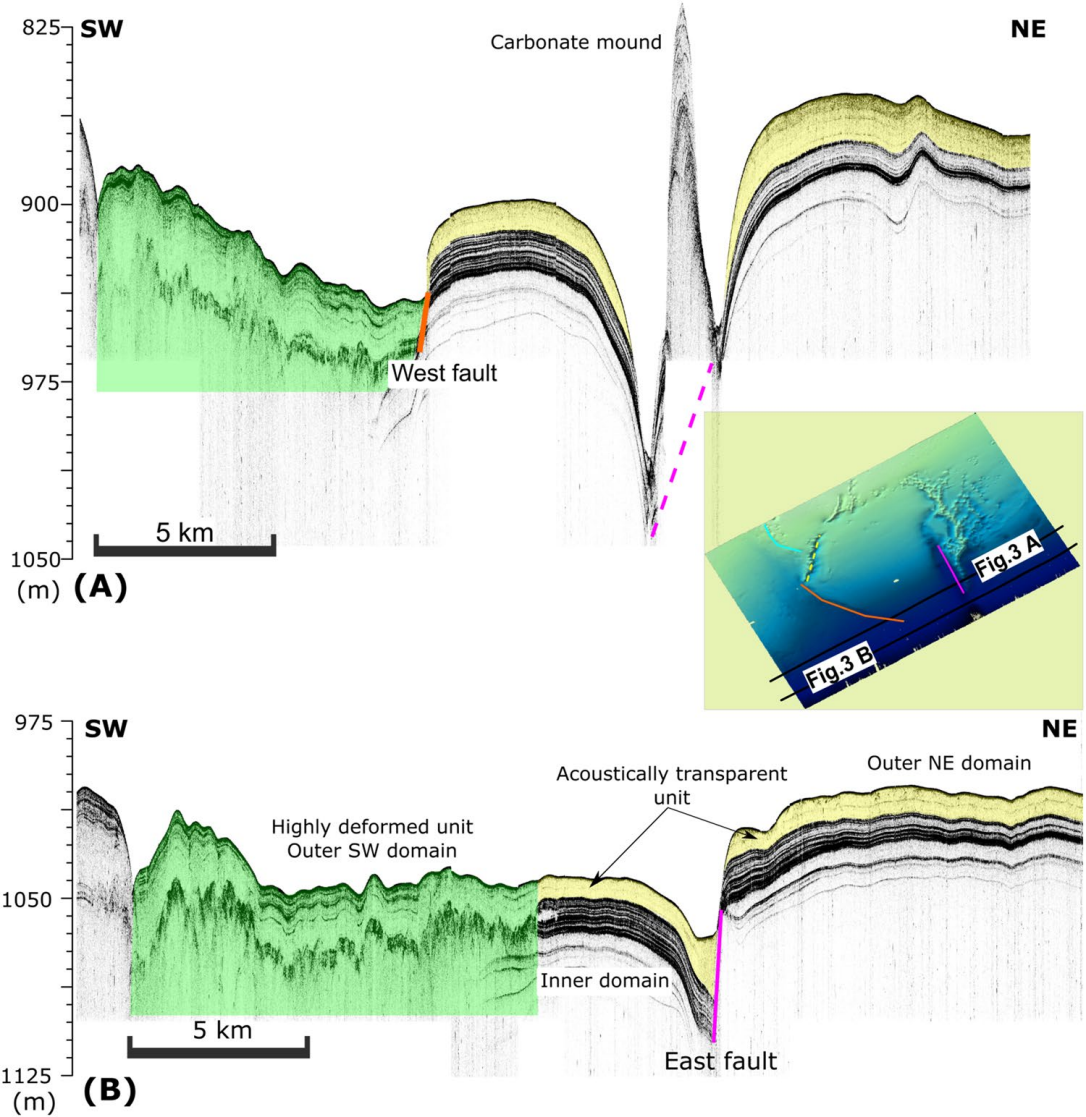
SW-NE-trending single-channel CHIRP seismic reflection profiles showing the three morphologic domains: outer SW, inner, and outer NE. The outer SW is deformed while the inner preserves two main units: a basal veneer unit and an upper transparent unit. The carbonate mound in (**A**) is emplaced along the strike of the west fault, as shown in. (**B**) Interpretation made with SonarWiz software, version 7.04 (CHESAPEAKE TECHNOLOGIES; https://chesapeaketech.com).

The study area is divided into three main domains (inner, outer SW, and outer NE) according to the sub-surface seismic facies pattern and the occurrence of shallow faults that may be related to the mounds and that are used to establish the domain boundaries (Fig. 3).

**Figure 4:**
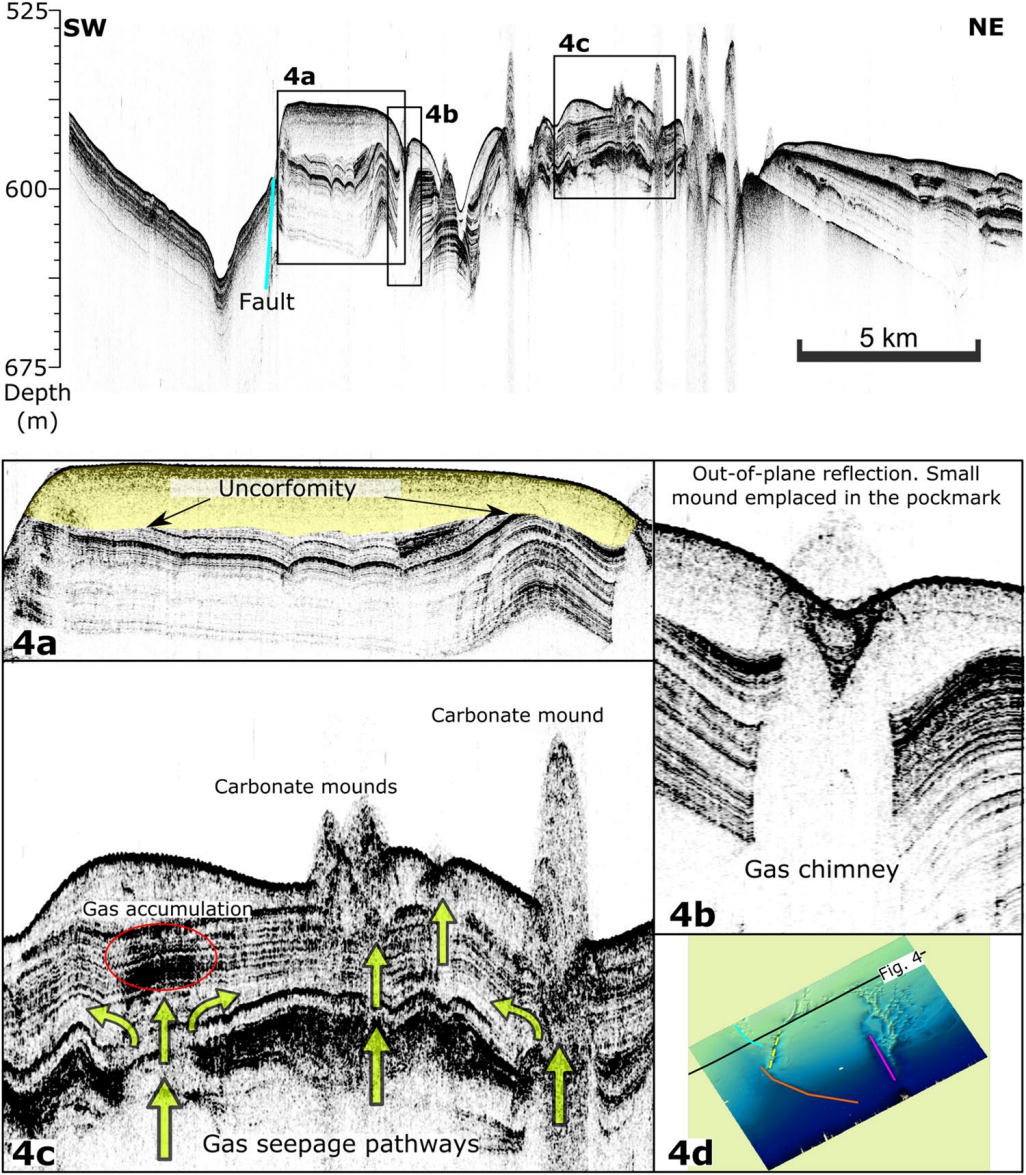
Above: Single-channel CHIRP seismic reflection profile of the NW boundary. Insets below: (**a**) Uplifted block showing the deposition unconformably over a folded layer. (**b,c**) Gas-related features affecting the upper sedimentary units in the inner domain: pockmarks, carbonate mounds, chimneys, and acoustic disturbances. Location map of chirp seismic line. (**d**) Both interpretation and map made with SonarWiz software, version 7.04 (CHESAPEAKE TECHNOLOGIES; https://chesapeaketech.com).

The inner domain comprises a < 60 m thick package, and it is bounded to the southwest by a normal fault (the east fault in Fig. 3A). An acoustically transparent upper unit lies at the top of this package. This unit thickens northwards from 8 to 22 m (Figs. 3A,B and 4).

A WNW-trending fault (the west fault) represents the boundary between the inner and outer SW domains. The vertical throw across this fault decreases towards the ESE from 60 to 20 m (Fig. 3A,B). The hanging wall comprises a slumped block in which the veneer layer appears diffuse and thin, and the internal reflectors and seafloor relief look irregular, unlike the soft relief of the inner domain (Fig. 3A,B). To the northwest along the fault strike, this boundary deflects to the northeast in the SW extremity of the narrow mounds sector of the ACCR (yellow dashed line in Fig. 3 map inset).

The NW limit of the ACCR (Fig. 4) shows remarkable differences with the rest of the area. In the outer SW domain, the characteristics of both the outer SW and inner domains are present, i.e., a fault bounding an uplifted block and a slump in the hanging wall. However, unlike the inner domain, the transparent unit lies unconformably over a folded veneer layer (Fig. 3, inset 1). In the inner domain, the upper sedimentary units are interrupted and deformed by growing mounds and other gas-related features, such as pockmarks and chimneys (Fig. 4B,C).

**Figure 5:**
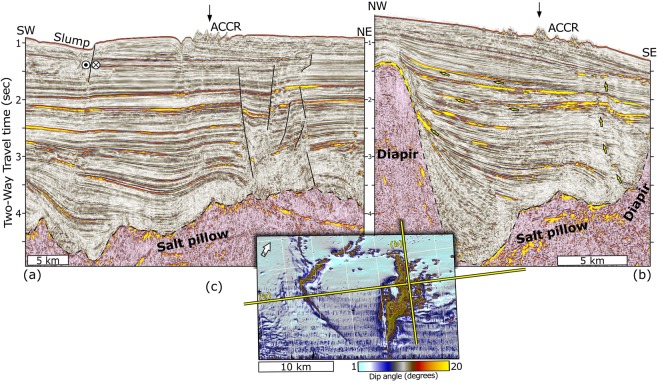
Deep multichannel seismic reflection profiles presented as amplitudes overlain by the root mean square (RMS) attributes of the amplitude (yellow hues). The slump shown in (**a**) corresponds to the gravitational deformation observed in the upper SW sedimentary layer in Figs. 3 and 4. Bright spots and high RMS values suggest possible gas locations. Arrows indicate gas pathways (**b**). (**c**) Slope map of ACCR location and seismic lines corresponding to profiles 5a and 5b. Interpretation performed with Petrel-Schlumberger, academic version (https://www.software.slb.com/products/petrel).

**Figure 6:**
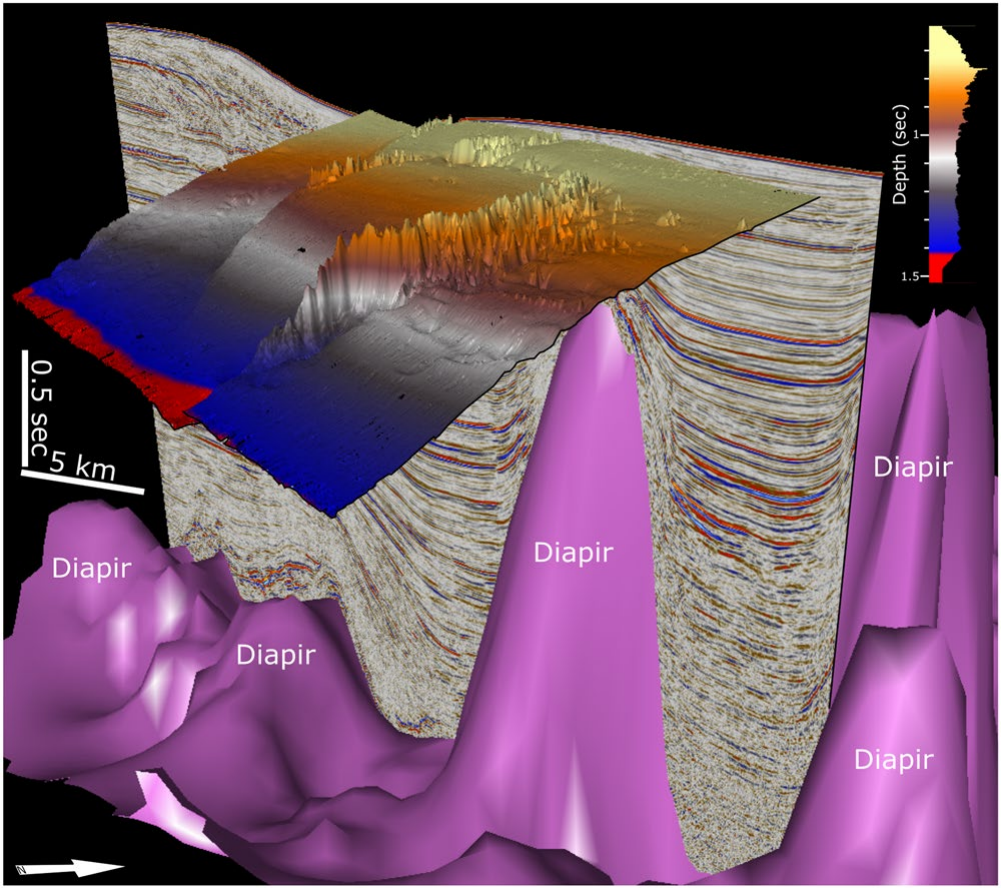
3D view of the spatial relationship between salt diapirism and the morphology of the ACCR. Interpretation performed with Petrel-Schlumberger, academic version (https://www.software.slb.com/products/petrel).

Multichannel seismic data present a spatial correlation between the ridge and salt diapirs (Figs. 5 and 6). This relationship occurs via faults and fractures that rise from the diapirs and extend up to the present seafloor. The data show the relationship among salt diapirs (pink hues), sediment layer fracturing, and the ascent of fluids in the area of the ACCR. The slump shown in profile (a) corresponds to the gravitational deformation observed in the upper SW sedimentary layer in Figs. 3 and 4. Deep-rooted fractures and faults, which are more abundant in the NE half, profile (a), could explain the eastward-scattered mounds observed in Fig. 2B.

Box-core samples (Supplementary Material 1) revealed abundant living and dead deep-water ramified scleractinian corals living on top of the ridge. These included the reef-building species *Lophelia pertusa*, *Enallopsammia rostrata*, and *Madrepora oculata*. These corals were home to a large number of associated fauna, including sponges, ophiuroids, asteroids, crinoids, and bryozoans. The solitary scleractinian coral *Caryophyllia diomedeae* was also present. Within the sediment, a large number of polychaete worms were present and represented most of the benthic macrofauna.

## Discussion

The newly discovered ACCR exhibits a structure, composition, and dimensions equivalent to mound complexes in the North Atlantic^2,15^ and can be considered a giant carbonate ridge. In particular, its outstanding heights above the surrounding seafloor and its kilometre-scale lateral continuity make the ACCR an equivalent counterpart of the giant carbonate mounds identified in the NE Atlantic Ocean carbonate mound province off Ireland (e.g.^2,3,5,6^). Since similar major carbonate mounds have been interpreted as CWCs, not only in the carbonate mound province off western Ireland but also in other settings in the NE Atlantic Ocean and the Mediterranean Sea, we propose a similar origin for the ACCR. Indeed, the carbonate complex is home to vulnerable marine ecosystems composed of CWCs associated with a rich assemblage of benthic invertebrate fauna (sponges, echinoderms, bryozoans, among others). These pieces of evidence support the interpretation of the ACCR as a western analogue of the large carbonate mounds found in the eastern Atlantic Ocean.

It is widely assumed that the major development and growth of CWC mounds is mainly fostered by favourable hydrodynamic conditions and by nutrient availability^1,2^. However, there is considerable controversy regarding the role played by fluid flows in carbonate mound formation and development. For example, some studies have suggested an association between major carbonate mound development and fluid flow occurrences (e.g.^5,54,55^) because seepage provides a substantial and reliable food source for bacteria, which are part of the food chain of higher organisms. In some cases, the fluid flow occurrences seem to foster the initial development of the mounds^6^, as fluid flow can help form the hard substrates necessary for the subsequent establishment of colonies^9^. In many other cases, however, this relationship has remained relatively obscure and unproven^3,10^, due largely to post-depositional alteration^2^. This study strongly supports the occurrence of a close relationship between the upward displacement of gas/fluids associated with faults and fractures generated by the ascending movement of a salt diapir and the spatial arrangement of the features comprising the ACCR (Fig. 1). Notably, this spatial correlation occurs in the Albian Gap, a domain characterized by a paucity of salt structures in comparison to the abundance of such structures in the deeper São Paulo Plateau. Therefore, the ACCR constitutes a rather singular regional feature influenced by a focused and rather localized occurrence of fluid flows.

The halokinesis beneath the ACCR plays a crucial role in facilitating the ascent of gas/fluids towards the ocean floor. Salt domes, walls, and other kilometre-scale amorphic volumes progressively change shape in the sub-surface while deforming the adjacent stratigraphy. Their ascent as salt diapirs extends and fractures the overlaying stratigraphy while tilting the dome flanks above the sagging rim of the diapir^23^. These modifications create new fluid flow pathways, and consequently, hydrocarbon gases and fluids flow along the flanks and through the fractures up the newly formed dip towards the diapir^56^. Additionally, the local structures and stratigraphy dictate the lateral, slanted, and vertical gas/fluid migration along paths with higher permeability^23,33^, while diapirism fractures the overlying succession and creates additional vertical upward migration paths.

These migrating paths may result in gas/fluid leakage through the seafloor (Figs. 5 and 6). In that sense, shallow gas expressions, such as vertical blanking, which is indicative of gas chimneys topped by V-shaped seafloor pockmarks, indicate recent to sub-recent active seepage (Fig. 4). One of the potential fluids escaping from the seafloor is methane^57^. Methane is acquiring increasing importance in the study of continental margins because it is a greenhouse gas and is considered an energy resource. Methane expulsion from the sub-surface can promote seafloor instabilities and geohazards. One of the potential instabilities appears as the east-trending slump bounding the SW margin of the ACCR (Fig. 3A,B).

As mentioned before, the physical environment around carbonate mounds seems to be critical for its long-term development. Taking into account the magnificent dimensions of the ACCR, we may speculate upon the occurrence of favourable physical conditions for its continuous development. Many large carbonate ridges appear to be closely associated with density interphases at water mass boundaries due to the occurrence of mixing processes^1,2,4^. Other studies suggest the importance of bottom current velocities and intensities^3,11,58,59^, leading to the interpretation that high oxygenation levels^13,60^ are the main forcing agents favouring the development of mounds. In the study area, ventilation processes in the thermocline between the SACW and the AAIW^61^ could be induced by mesoscale eddies of the BC and could favour the occurrence of mixing processes.

Additionally, the ACCR would have been affected by a persistent strong flow (<0.3 m.sec^−1^) of the IWBC^42,46^ that intensifies towards the northeast^62^, transporting cold AAIW. The size of the mounds and their long-term build-up may also be dependent on (1) the amount of sediment input driven by currents^11,63^; (2) the mound capacity for sediment baffling^10^; and (3) the enhancement of productivity conditions eventually leading to higher food availability^58,64,65^. In this sense, the basinward-dipping ACCR bulge, which is several metres above the average surrounding bathymetry, can attest to the ridge’s capacity to trap sediments. In this setting, the main reef-building coral species found in the study area likely used the higher ground to benefit from stronger currents and, consequently, a larger food supply, as documented in other carbonate mounds formed by corals^59^.

Once formed, the ACCR must have acted as a significant topographic obstacle, as documented in other carbonate mound provinces^1^ (Lo Iacono *et al*., 2018). The occurrence of comet-like marks and elongated channels, the asymmetrical morphology of the flanks of the ridges, and the truncation of seismic reflections all support the existence of a disturbing effect of the ACCR on the regional bottom flow patterns. Thus, the rough carbonate mound morphology channel would divert the bottom flows along the northern and western boundaries of the complex, forming the elongated erosional depressions that follow the flanks of the mounds. Northeast of the complex, individual mounds force the bottom currents to form downstream-facing comet-like marks^66^.

We highlight the uniqueness of the ACCR, which is the first giant carbonate ridge described across the SW Atlantic margin and is fed by fluids escaping from the sub-surface via channels along salt tectonics-related faults and fractures. Its location above a prolific hydrocarbon province, which is abundant in evaporites, and its direct association with other types of gas/fluid escape features (pockmarks, chimneys, and depressions), make this particular complex a worldwide example for deep-water hydrocarbon-associated carbonate mounds.

The ACCR represents a modern analogue to other similar, less accessible buried structures and can provide a modern case study for understanding similar structures that developed in the geological past but are now covered by overlying strata. From a biological perspective, the ACCR is a very important structure because it harbours vulnerable marine ecosystems (VMEs) that require conservation due to their importance to both deep-sea and global biodiversity^65^. These corals serve as refugia for a myriad of invertebrates seeking food, protection, and reproductive ground^15^ and are extremely important for the ocean carbon cycle.

## Methods

This study is based on recently acquired multibeam bathymetry and single-channel CHIRP data collected aboard the R.V. Alpha Crucis during January-February 2019 (Fig. 1) (Supplementary Material 2). Multibeam data were collected using a 512-beam Teledyne Reson Seabat 7160 with a transducer frequency of 44 kHz. A maximum beam angle of 110° was used, with a 50% overlap between ship tracks. Sound velocity profiles were acquired using an AML Oceanographic Sound Velocity Probe at the beginning of each multibeam line.

The bathymetric data were processed using sound velocity correction, filtering, and mosaicking with Sonarwiz Software v. 7.02 (CHESAPEAKE TECHNOLOGIES, USA). A 30 × 30 m pixel size bathymetry map was generated. Single-channel seismic-reflection CHIRP data were obtained using a Knudsen 3260 operating at 3.5 kHz. The data were collected together with the multibeam survey. The.keb Knudsen’s proprietary files were transformed into standard.sgy files and post-processed using Meridata MDPS software. Finally, sediment samples were collected using an Ocean Instruments BX-650 box corer. Benthic fauna from these corers was sorted on board for larger organisms and in the laboratory under a stereomicroscope. Specimens were identified to the lowest possible taxonomy level and kept in 70% ethanol for long-term storage.

Deep 2D time‐migrated multichannel seismic reflection profiles were provided by the Brazilian National Oil Agency and were analysed using Petrel-Schlumberger software. The positive polarity profiles presented are part of a cumulative 19,100-km-long dataset extending along the entire Brazilian continental margin^38^, covering an area of 562,000 km^2^. Data were collected during six surveys since 2000 in profiles extending down to 7, 8, 9, 10 and 12 s two‐way travel‐time (TWT) with a sample rate of 4–8 ms, a fold of 50–109, and vertical resolution of a few metres per reflector in the studied interval.

## Supplementary information

Supplementary Material

Supplementary Material
