# Supplementary figures and images for: The Alpha Crucis Carbonate Ridge (ACCR): Discovery of a giant ring-shaped carbonate complex on the SW Atlantic margin
Source: Sci Rep. 2019 Dec 10;9:18697. doi: 10.1038/s41598-019-55226-3 (PMC6904621; doi:10.1038/s41598-019-55226-3)

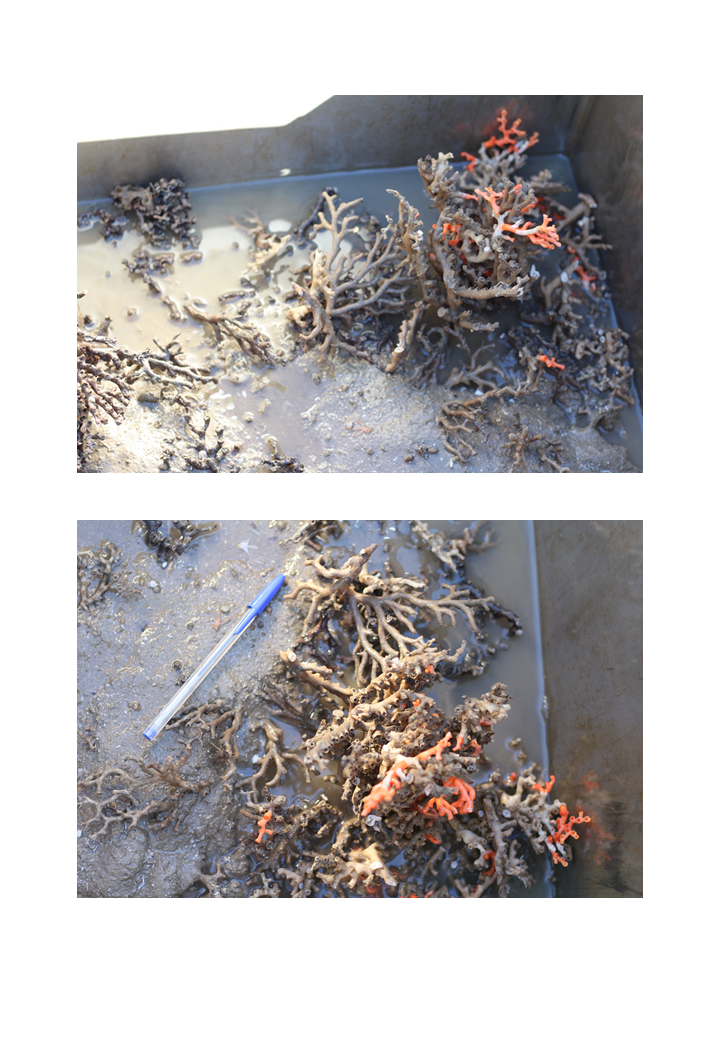

Supplement: Supplementary file 1 — Supplementary Material [file 41598_2019_55226_MOESM1_ESM.tif]
